# Minimally Invasive Management of Iatrogenic Ureteral Injuries with Ureteroscope Facilitated by Holmium Yttrium-Aluminum-Garnet Laser

**DOI:** 10.1155/2014/307963

**Published:** 2014-11-09

**Authors:** Zongyao Hao, Li Zhang, Jun Zhou, Xiansheng Zhang, Haoqiang Shi, Yifei Zhang, Song Fan, Chaozhao Liang

**Affiliations:** Department of Urology, The First Affiliated Hospital of Anhui Medical University and Institute of Urology, Anhui Medical University, Hefei 230022, China

## Abstract

The ureter is vulnerable during general, gynecologic, and urologic surgeries. The traditional open surgical approaches to treating the iatrogenic ureteral injuries have shown several disadvantages such as relatively high rate of severe complications. Although the applications of endourological techniques for management of lower ureteral strictures have been routinely introduced over the last 10 years, most of the different modalities were based on the utilization of hydrophilic instruments that can facilitate the traversal of strictures surrounded by the sutures with gradually increasing force. Interestingly, we have revealed the Ho:YAG laser as the outstanding auxiliary approach to incising the sutures during the ureteroscopy for its well-controlled penetration depth, minimal scarring, and precise cutting. As far as we know, the combined utilization of Ho:YAG laser to incise the sutures responsible for the strictures and double J ureteral stent for drainage has not been extensively reported. Normal ureters of the patients managed by this novel approach were shown by the follow-up 3-4 months later, which demonstrated that the available technique was promising to effectively treat the iatrogenic ureteral injuries.

## 1. Introduction

Despite the ongoing advances in modern surgical techniques, the unexpected iatrogenic ureteral injuries still represent one of the most serious postoperative complications in intra-abdominal, retroperitoneal, or pelvic operations with significant morbidity due to the possible impairment of renal function and susceptibility to microbial pathogens [1, 2]. The injuries which have a documented incidence of 0.3% to 1.5% [3] are associated with accidental ligation, laceration, crushing, stretching, and devascularization [4]. The surgical interventions responsible for iatrogenic ureteral injuries include the gynaecological surgeries such as total abdominal hysterectomy, bilateral salpingo-oophorectomy followed by radical hysterectomy [5] and also consist of the colorectal and urological operations; in such procedures the ureters run close to the field of dissection [6, 7]. Generally, iatrogenic lower ureteral injuries are caused by the aberrant sutures responsible for the strictures or obstructions and subsequently repaired utilizing end-to-end ureteral anastomosis or a Boari flap with ureteral reimplantation or some combinations, as well as a psoas hitch [8, 9]. However, the above-mentioned traditional open surgical techniques are somehow no longer widely accepted as options for the management of iatrogenic lower ureteral injury, as some reports have associated ureteroureterostomy with a relatively high rate of severe complications, although the issue is still controversial [10, 11]. Besides, another reason for ureteroureterostomy rarely being introduced to repair lower ureteral injury was that distal ureters had a relatively poor vascular and blood supply while the pelvic ureter appears to have a high preponderance of plexiform vessels, which has been considered susceptible to ischemia after transection [12]. Thus, it is highly desirable to seek the alternative treatment approach. Fortunately, laparoscopic reconstructions of ureteral injuries have been introduced in the clinical practice, and the experience is continuously extending [13], among which, endoscopic minimally invasive approaches increasingly emerge to be applicable options for the management of ureteral lesions. Several cases of iatrogenic ureteral injuries successfully managed by percutaneous nephrostomy were reported [14], whereas various modalities applied to reconstructions of ureteral injuries still have many disadvantages to overcome, such as hemorrhage and the risk of damage to surrounding tissues. Here, we report an ideal, novel, available, and also minimally invasive technique with combined utilization of the ureteroscope and Ho:YAG laser to manage the ureteral injuries and related obstructive symptoms sustained during surgeries, where injuries and strictures generally are caused by the inappropriate suture passing through or surrounding the ureter.

## 2. Case Presentation

From October 2009 to May 2013, 12 patients (11 females and one male with average age of 48.8 years and range from 37 years to 68 years) had been postoperatively detected to have iatrogenic lower ureteral injuries sustained during general, gynecologic, and urologic procedures. These patients (9 left side ureteral lesions, 3 right side ureteral lesions; 8 cases of iatrogenic ureteral injury occurred in radical hysterectomy, 3 cases occurred in resection of rectal cancer, and 1 occurred in the radical resection of uterine recurrent tumor, Table 1) presented to our department with the postoperative symptoms related to hydronephrosis and/or compromised renal function.

**Figure 1:**
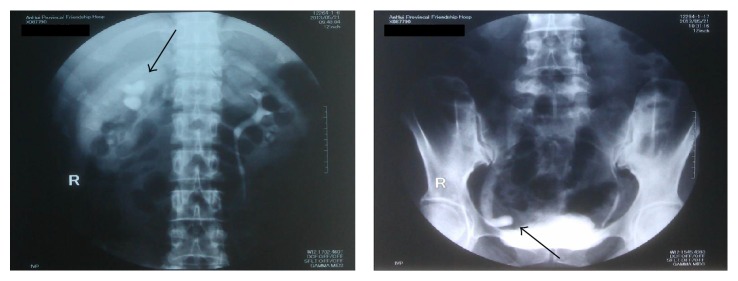
A 39-year-old female patient who had iatrogenic ureteral injuries during hysterectomy. The standard intravenous pyelography showed the pelvicaliceal dilatation and hydronephrosis in the obstructed kidney and precise location of the obstructed ureter (indicated as the black arrows).

These symptoms included low-grade fever, chills, flank, loin, back and/or abdominal pain, urinary leakage, and increased urine leakage from drainage tube. Routine blood chemistry examinations showed no or slightly increased concentrations of blood urea and creatinine. The marked pelvicaliceal dilatation and hydronephrosis in the obstructed kidney (so-called pale kidney) and also precise location of the obstructed/injured segment were observed via the standard intravenous pyelography facilitated by contrast agents (Figure 1). No hypersensitivity to drugs and normal blood coagulation function were guaranteed. After administration of conscious sedation, patients received the operations carried out by the same surgeon.

**Figure 2:**
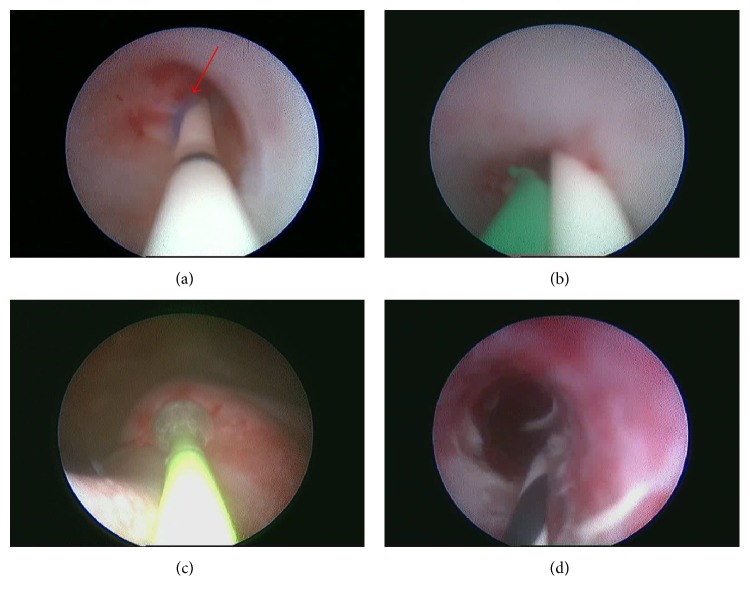
Step-by-step illustration of minimally invasive management of iatrogenic ureteral injuries with combined ureteroscope and Ho:YAG laser. The unexpected sutures were indicated as the red arrow in the upper left ureteroscopic images. After Ho:YAG laser incision (upper right image) and balloon dilation to an adequate diameter of the ureter (lower left image), the obstruction of the ureter caused by the aberrant sutures was completely relieved (lower right image).

We employ the procedures of combined utilization of the ureteroscope and Holmium laser to manage the iatrogenic ureteral injuries (one representative case) as follows. The first step was the retrograde insertion and placement of a ureter catheter (3Fr) in the ureter to the location of the obstruction to increase support. Then, a 8/9.8Fr rigid ureteroscope passed the ureter to the location of the obstruction under the guide of catheter. When the distal location of the stricture with part or complete sutures ligation was visualized, a stiff 365 *μ*m Ho:YAG laser optical fiber was placed at the precise location of the obstruction and subsequently the Ho:YAG laser at 3.0 J power/10 Hz was applied to incise the sutures surrounding the stricture segment (Figure 2). This procedure was followed by the removal of the sutures via the foreign body forceps, and the site of obstruction was gradually dilated with a classic X-Force U30 ureteroscopic balloon dilation to an adequate diameter of 6 mm. Under the support of 0.032-inch zebra guidewire (Urovision), a standard Bard-InLay internal 6F double J ureteral stent was placed (Figure 2).

**Figure 3:**
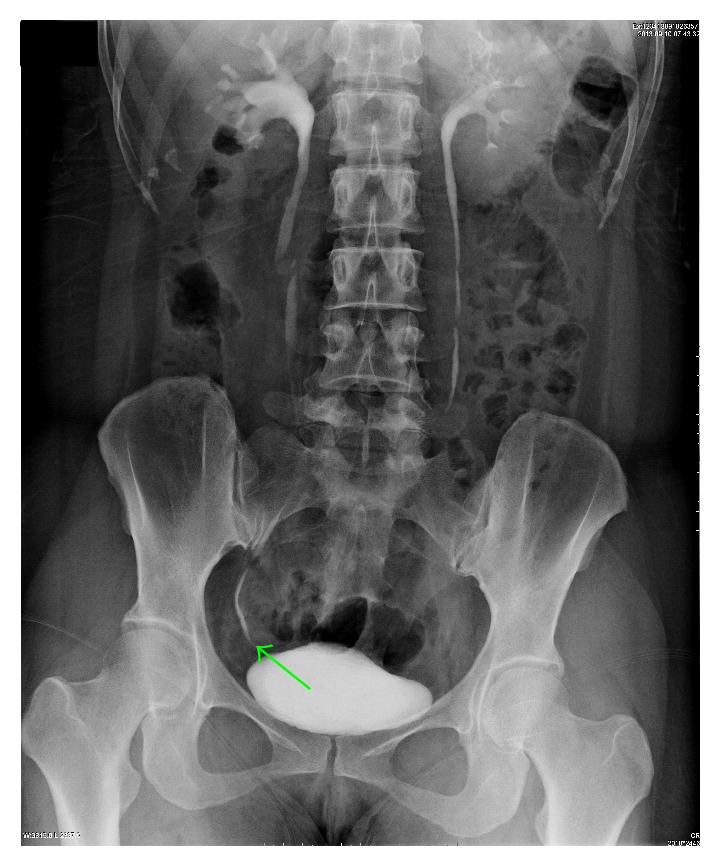
Follow-up for the patients posttreatment of 3 months. After removing the double J stent, the standard intravenous pyelography showed the normal right ureter with no obstruction or stricture (indicated as the green arrow). Meanwhile, hydronephrosis in the originally obstructed right kidney was largely or completely reversed.

Follow-up for the patients posttreatment of 3-4 months showed the normal ureters with no obstruction or stricture by intravenous pyelography after removing the double J stent. Meanwhile, hydronephrosis in the originally obstructed kidney was largely or completely attenuated (Figure 3). Eventually, the available technique was well established.

## 3. Discussion

The ureter is a vulnerable anatomic structure which can be easily compromised in the retroperitoneal space during general, gynecologic, and urologic surgeries. The traditional open surgical techniques such as end-to-end ureteroureterostomy with or without psoas hitch or Boari flap for the iatrogenic ureteral injuries have shown the obvious drawbacks of relatively high rate of severe complications and poor vascular blood supply in the distal ureters [15]. The increasing applications of endourological approaches to treating lower ureteral strictures have become routinely techniques over the last decade. Currently, various modified techniques have been introduced to incise ureteral strictures based on the utilization of hydrophilic apparatus that can facilitate the traversal of strictures, whereas classic balloon dilation management cannot solve hemostasis problem, as well as the electrocautery techniques which might lead to adjacent tissues damage [16]. Surprisingly, in our clinical practice, the Ho:YAG laser has been revealed to be an outstanding instrument for incising the sutures during the ureteroscopy, as the penetration depth of the energy is under well-control; besides minimal scarring and precise cutting are also valid. A few studies have reported series of approaches such as using antegrade and retrograde to incise or eliminate the ureteral strictures [16, 17]. However, to the best of our knowledge, the combined application of ureteroscope, Ho:YAG laser, and balloon dilation to incise and simultaneously remove the sutures responsible for the strictures or obstructions has not been previously elucidated. This communication illustrates that lower iatrogenic ureteral injury with strictures or obstructions caused by the aberrant sutures can be effectively managed by minimally invasive endourological approaches.

## Figures and Tables

**Table 1:** Preoperative patients characteristics.

Procedure	Number of patients	Lateral		Age (yrs)
		Left	Right	
Radical hysterectomy	8	6	2	48.4 (37–59)
Radical resection of rectal carcinoma	3	3	0	50.1 (41–68)
Radical resection of uterine recurrent tumor	1	0	1	46

Total	12	9	3	48.8
